# Postoperative empyema complicated with descending necrotizing fasciitis: a case report

**DOI:** 10.1186/s40792-022-01492-9

**Published:** 2022-07-15

**Authors:** Yuka Kadomatsu, Harushi Ueno, Masaki Goto, Naoki Ozeki, Shota Nakamura, Toyofumi Fengshi Chen-Yoshikawa

**Affiliations:** Department of Thoracic Surgery, Nagoya University Graduate School of Medicine, 65 Tsurumai-cho, Showa-ku, Nagoya, 466-8550 Japan

**Keywords:** Empyema, Necrotizing fasciitis, *Streptococcus anginosus*

## Abstract

**Background:**

Empyema is a serious complication of lung surgery.

**Case presentation:**

We report a case of *Streptococcus anginosus* empyema complicated by descending necrotizing fasciitis after surgery. Ten days after the initial surgery, the patient presented with arrhythmia and hypotension with septic shock. Pleural fluid analysis led to the diagnosis of empyema, and surgical debridement (second surgery) was performed. One week after the emergency surgery, a palpable pink erythematous skin change was observed on the ipsilateral iliac crest. Hence, the second emergency surgery (third surgery) was performed to debride the subcutaneous and intramuscular abscesses.

**Conclusions:**

The possibility of descending abscess and necrotizing fasciitis should be considered when *Streptococcus anginosus* is detected.

## Background

The incidence rate of empyema after lung resection ranges from 2 to 16% [1, 2]. Antibiotics, properly timed thoracic drainage, and surgical intervention are the cornerstones of the treatment of empyema [3]. Although subcutaneous penetration of the abscess has been reported in cases of empyema due to serious infectious diseases, postoperative empyema with a descending subcutaneous abscess on the intramuscular layer and necrotizing fasciitis is rare. Here we report the case of a patient with *Streptococcus anginosus* empyema with a descending subcutaneous abscess and necrotizing fasciitis, which was successfully treated with an additional skin incision and multiple sessions of emergent debridement. This case suggests that not only the chest but also the surrounding skin should be monitored in patients with postoperative empyema; moreover, these patients might develop descending subcutaneous abscess and necrotizing fasciitis when *Streptococcus anginosus* is detected.

## Case presentation

**Fig. 1:**
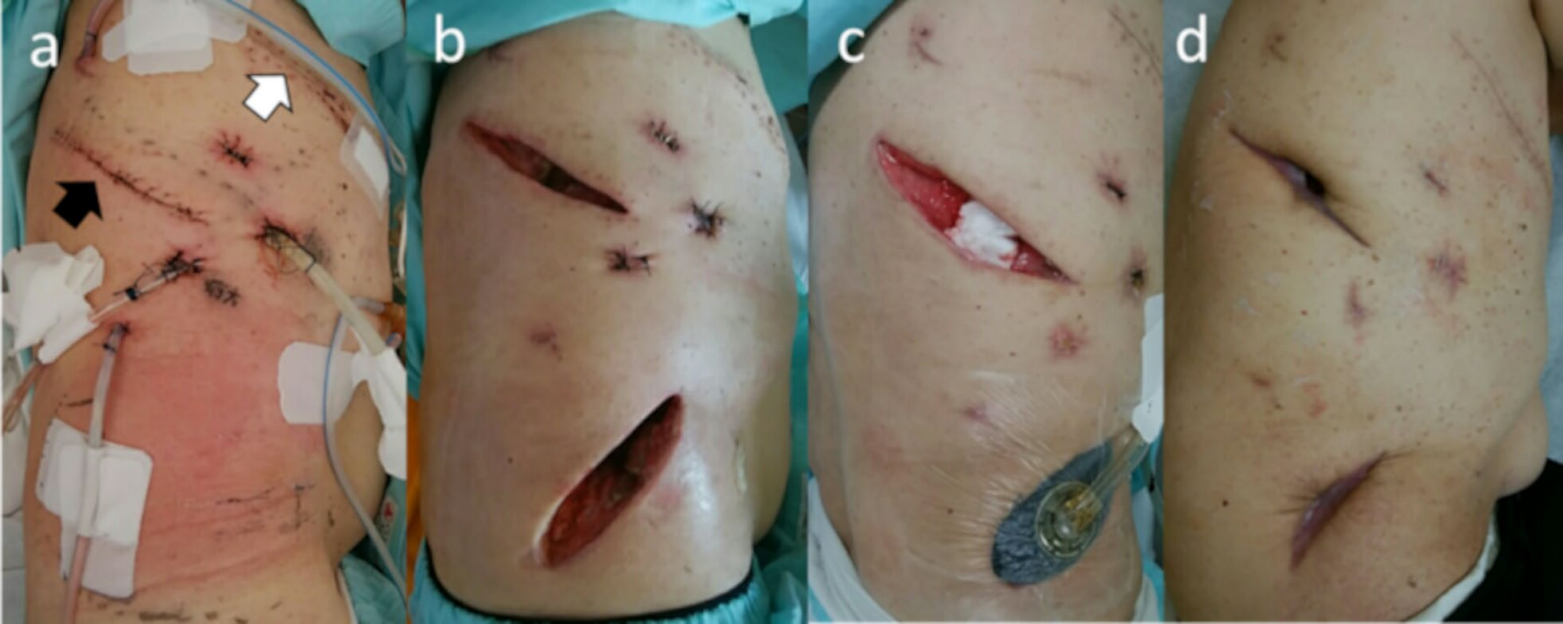
Appearance of the wound. **a** Just before the second emergency surgery. Redness can be observed at a considerable distance from the initial lobectomy wound (white arrow) or the first emergency open debridement wound (black arrow). **b** One month after the initial surgery. **c** The chest wound is treated with gauze exchange and washing, and the buttock wound was treated with negative-pressure therapy. **d** Four months after the initial surgery. The wound is completely closed without fistula

**Fig. 2:**
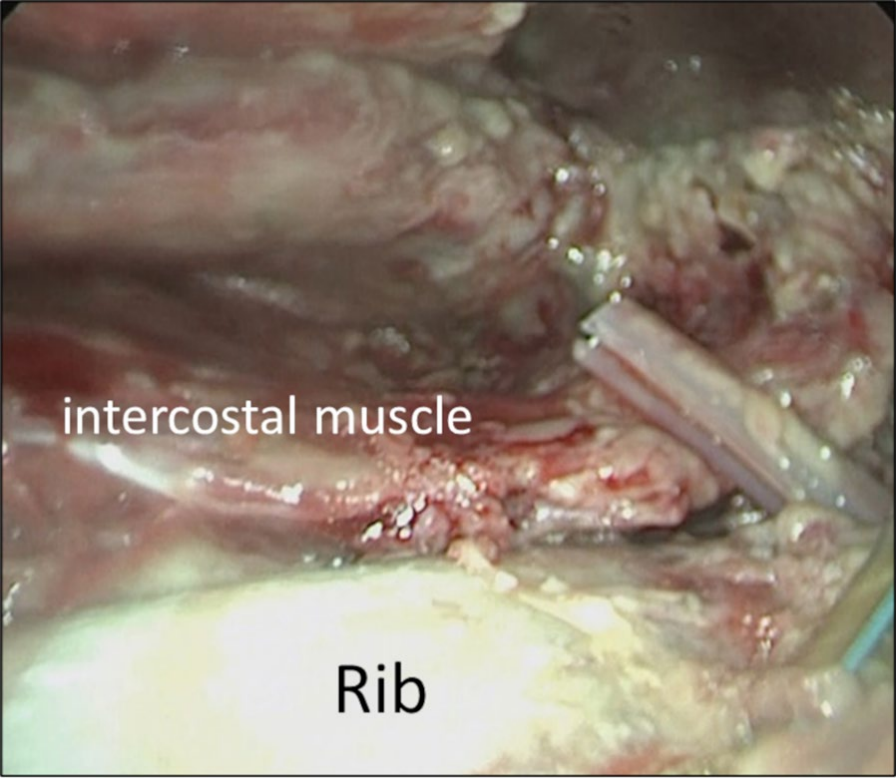
Adhesion of the pus can be clearly observed between the intercostal and serratus anterior muscles

A 74-year-old man presented with a nodule in the right upper lobe on radiographic scan during a pneumonia treatment. The patient had suffered from bacterial pneumonia caused by Streptococcus pneumoniae. Following piperacillin/tazobactam administration, the pneumonia improved itself. He had been diagnosed with adenocarcinoma through a transbronchial biopsy. His medical history included chronic obstructive pulmonary disease, severe emphysema, and diabetes mellitus. His diabetes was controlled with a single dipeptidyl peptidase-4 inhibitor, and his preoperative hemoglobin A1c level was 6.4%. Preoperative blood samples revealed no abnormalities and no symptoms of an inflammatory reaction. The surgical plan was thoracoscopic right upper lobectomy. A right upper-middle bilobectomy was performed to control the massive air leakage from the residual lung parenchyma. The approach was a lateral thoracotomy, and the serratus anterior muscle was split and spared as indicated by the white arrow in Fig. 1a. Prophylactic antibiotics were administered until the first postoperative day. On the third postoperative day, the patient underwent OK-432 pleurodesis because of a persistent air leak. On the 5th day after onset, the leak improved, and the thoracic drain was removed. The patient complained of loss of appetite but did not have a fever exceeding 37 °C. On the 10th postoperative day, the patient exhibited atrial fibrillation and hypotension. The contrast-enhanced computed tomography findings revealed fluid accumulation in the chest cavity, and intrathoracic abscess was suspected. Thoracocentesis of the right pleural effusion yielded white gray pus-like fluid with a pungent odor. Gram stain of the pleural effusion indicated a polymicrobial infection, including numerous gram-positive cocci in chains and gram-positive and gram-negative bacilli with phagocytosis. The pleural effusion culture yielded *Streptococcus anginosus*, *Prevotella oris*, *Porphyromonas* species, and *Gemella* species. Meropenem antibiotic was used for the infection. On day 11, a second surgery was performed for open debridement and intrathoracic lavage. From the day following the second operation, pus persistently drained again, and *Streptococcus anginosus* was isolated from the pleural effusion. The fever and inflammatory reactions showed improvement with antibiotics; however, on day 18, redness and warmth were evident in the buttocks ipsilateral to the surgical site (Fig. 1a). A third surgery was performed because of the suspicion of a subcutaneous abscess. The skin incision was made just above the most reddened area of the buttocks and split along the muscle bundles of the external oblique abdominis muscle. Pus adhered to the fascia, and necrotic fascia was observed. Debridement was performed, and the initial debridement wound revealed similar findings (Fig. 2). The chest and buttock abscess wounds were debrided, and both wounds remained open for infection control (Fig. 1b). *Streptococcus anginosus* was isolated from the necrotized fascia. After the third surgery, both abscess cavities were treated with daily gauze changes, and the buttock wounds were treated with negative-pressure wound therapy (V.A.C.ULTA Therapy System, KCI USA, Inc., San Antonio, TX; Fig. 1c). Approximately 4 months after the second surgery, the wound was completely closed without surgical intervention, and the patient remained alive without any symptoms (Fig. 1d).

## Discussion

We found that the abscess of the postoperative *Streptococcus anginosus* empyema was not limited to the thoracic cavity but spread to the intramuscular layers and descended as if it were a necrotizing fasciitis.

Subcutaneous penetration of the empyema abscess is called empyema necessitans and reported in cases of empyema due to tuberculosis or aspergillus infection [4]. However, this case showed not only an abscess draining from the thoracic cavity but also a widespread fascial necrosis with relative preservation of the skin and underlying muscle. Culture of the necrotic fascia revealed the same *Streptococcus anginosus* group (SAG) as in the thoracic drain sample, which suggests a necrotizing descent fasciitis arising from the empyema.

SAG*,* previously known as the *Streptococcus milleri* group, are microaerophilic bacteria. The SAG bacteria reside in the oral and upper respiratory tracts and are the causative agent of necrotizing descent mediastinitis [5]. Thoracic infections associated with *Streptococcus anginosus* are known to be highly invasive and pyogenic, with a propensity for abscess and empyema formation [6]. Our patient had mild diabetes mellitus, relatively good glycemic control, and no other comorbidities that would indicate susceptibility to infection (Fig. 2).

## Conclusions

**Fig. 3:**
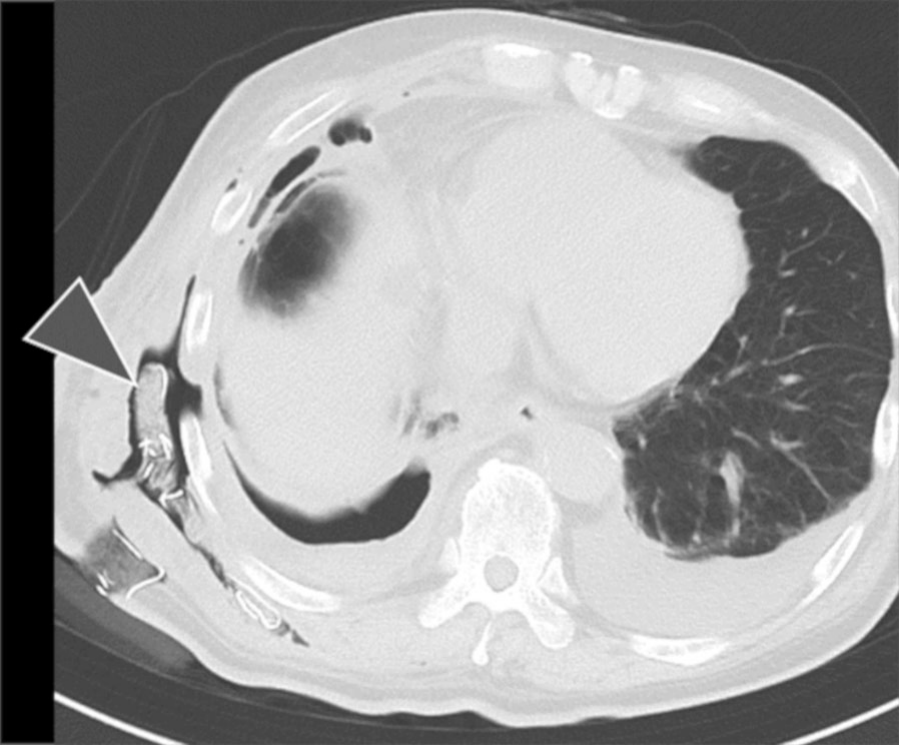
CT scan demonstrating bilateral pleural effusion and abscess cavity extending below the serratus anterior muscle. CT: computed tomography; Black triangle: abscess cavity

We experienced a successfully treated case of postoperative empyema with descending necrotizing fasciitis. SAG empyema can cause descending necrotizing fasciitis. When SAG is detected, careful attention should be paid to signs of rapid abscess expansion, and early debridement is essential for infection control (Fig. 3).

## Data Availability

The data that support the findings of this study are available from the corresponding author upon reasonable request.

## Abbreviation

SAG: *Streptococcus anginosus* Group
